# Review of the efficacy of infrared thermography for screening infectious diseases with applications to COVID-19

**DOI:** 10.1117/1.JMI.8.S1.010901

**Published:** 2021-03-25

**Authors:** Kosar Khaksari, Thien Nguyen, Brian Hill, Timothy Quang, John Perreault, Viswanath Gorti, Ravi Malpani, Emily Blick, Tomás González Cano, Babak Shadgan, Amir H. Gandjbakhche

**Affiliations:** a National Institutes of Health, Eunice Kennedy Shrive National Institute of Child Health and Human Development, Bethesda, Maryland, United States; b University of British Columbia, Vancouver, British Columbia, Canada

**Keywords:** infrared thermography, thermal camera, core body temperature, fever screening, coronavirus, infectious disease

## Abstract

**Purpose:** The recent coronavirus disease 2019 (COVID-19) pandemic, which spread across the globe in a very short period of time, revealed that the transmission control of disease is a crucial step to prevent an outbreak and effective screening for viral infectious diseases is necessary. Since the severe acute respiratory syndrome (SARS) outbreak in 2003, infrared thermography (IRT) has been considered a gold standard method for screening febrile individuals at the time of pandemics. The objective of this review is to evaluate the efficacy of IRT for screening infectious diseases with specific applications to COVID-19.

**Approach:** A literature review was performed in Google Scholar, PubMed, and ScienceDirect to search for studies evaluating IRT screening from 2002 to present using relevant keywords. Additional literature searches were done to evaluate IRT in comparison to traditional core body temperature measurements and assess the benefits of measuring additional vital signs for infectious disease screening.

**Results:** Studies have reported on the unreliability of IRT due to poor sensitivity and specificity in detecting true core body temperature and its inability to identify asymptomatic carriers. Airport mass screening using IRT was conducted during occurrences of SARS, Dengue, Swine Flu, and Ebola with reported sensitivities as low as zero. Other studies reported that screening other vital signs such as heart and respiratory rates can lead to more robust methods for early infection detection.

**Conclusions:** Studies evaluating IRT showed varied results in its efficacy for screening infectious diseases. This suggests the need to assess additional physiological parameters to increase the sensitivity and specificity of non-invasive biosensors.

## 1 Introduction

This paper reviews and summarizes existing information on the sensitivity, specificity, positive predictive value (PPV), and negative predictive value (NPV) of infrared thermography (IRT) utilized in screening for fever as well as the incidence of respiratory infectious diseases during a pandemic. The screening scale ranges from a small, well-controlled laboratory to a massive, uncontrolled airport. In addition, we discuss the advantages and limitations of IRT in detecting infection and suggest solutions for these limitations. Finally, we present options for the use of IRT toward the detection, diagnosis, and monitoring of coronavirus disease 2019 (COVID-19) during this ongoing pandemic.

### 1.1 Infectious Diseases, Symptoms, Early Screening

**Fig. 1:**
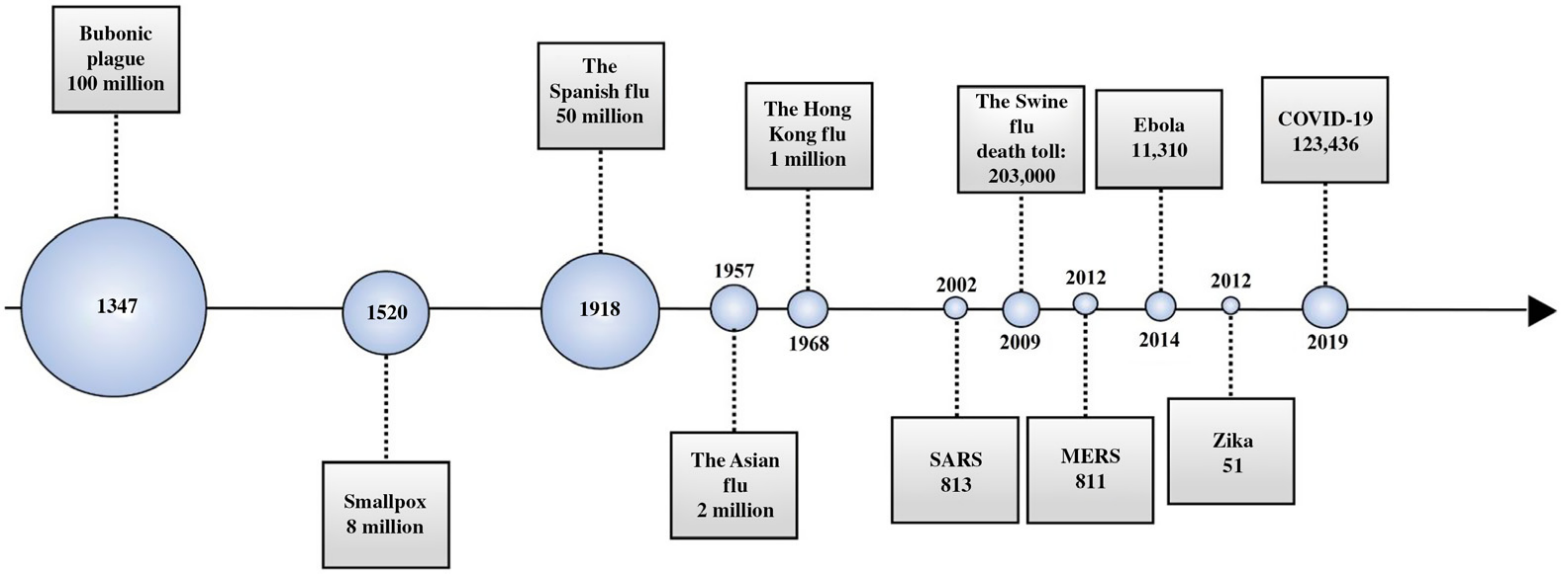
Timeline overviewing history and impact of major pandemics and their death tolls.^2^^,^^3^

Infectious diseases are caused by pathogenic microorganisms such as bacteria, viruses, parasites, or fungi that are spread, directly or indirectly, between individuals.^1^ The known pandemics throughout history have been related to bacteria and viruses (Fig. 1).^2^^,^^3^ Currently, the worldwide outbreak of the novel COVID-19 poses a significant threat to global health. The virus causing COVID-19, severe acute respiratory syndrome coronavirus 2 (SARS-CoV-2), is in the coronavirus family that caused the outbreaks of severe acute respiratory syndrome (SARS) and Middle East respiratory syndrome (MERS).^4^ As of June 15, 2020, SARS-CoV-2 has infected 7,963,453 people worldwide, killing a total of 434,388, numbers that will increase as the virus continues to spread.^5^^,^^6^

Symptoms of infectious diseases vary depending on the bacterial or viral agent; however, there are two common symptoms reported in many diseases. These are a fever and an increased antibody count in the blood.^7^ The diseases caused by the coronavirus family appear to result in similar symptoms, including fever, headache, dry cough, shortness of breath, gastrointestinal distress, and pneumonia (Table 1).

**Table 1:** Infectious diseases caused by coronavirus species.

Virus	Disease	Incubation period (days)	Symptoms	Infected people	Deaths	Fatality rate (%)
SARS-CoV	SARS	4.7 (95% CI 4.3 to 5.1)	Fever, headache, dry cough, shortness of breath, pneumonia, and gastrointestinal	8437	813	10
MERS-CoV	MERS	5.2 (95% CI 5.0 to 6.5)		2442	811	35
SARS-CoV-2	COVID-19	4.9 (95% CI 4.4 to 5.5)		7,963,453^a^	434,388^a^	

a Data collected on June 15, 2020;^5^ numbers continuing to rise.

Early screening and diagnosis of infectious diseases can prevent or reduce the spread of the diseases, improve the effectiveness of treatment options, and reduce health care costs.^8^ Healthcare workers who are repeatedly exposed to the disease are at high risk of getting an infection.^9^^,^^10^ The elderly, those possessing pre-existing health conditions such as chronic lung, heart, liver, kidney diseases, obesity, and diabetes, and those who are immunocompromised are at higher risk of a serious illness or life threatening infection.^11^ Considering the large groups of at-risk individuals, screening and early detection/diagnosis of the disease are crucial.

### 1.2 Fever Screening

Infrared thermometers or thermal cameras have been used extensively to screen febrile patients and travelers at the time of pandemic for non-contact and rapid monitoring of body temperature. Mass fever screenings have been performed in different places such as airports,^12^^,^^13^ ports (seaports),^14^^,^^15^ border (ground) crossings,^15^^,^^16^ and other public places such as hospital entrances. There is no strong evidence of the effectiveness of port and border crossing mass screening in delaying local transmission.^17^ Airport screening for fever was common at the time of pandemics.^17^^–^^19^ The intention was to identify people with high body temperature and stop them from travelling to reduce local transmissions. However, these infrared thermometers measure body surface temperature, which is not always a reliable surrogate for the core body temperature that is affected by infection.^20^ Infrared thermal cameras were usually mounted on a wall or ceiling to capture thermograms of the travelers’ faces.^21^ These cameras were not used on a daily basis but only at the time of epidemics or pandemics.^22^^,^^23^ Several studies examined airport screening to evaluate the effectiveness of the entry/exit screening along with the reliability of the thermograms.^13^^,^^18^^,^^19^

### 1.3 Infrared Thermography

**Fig. 2:**
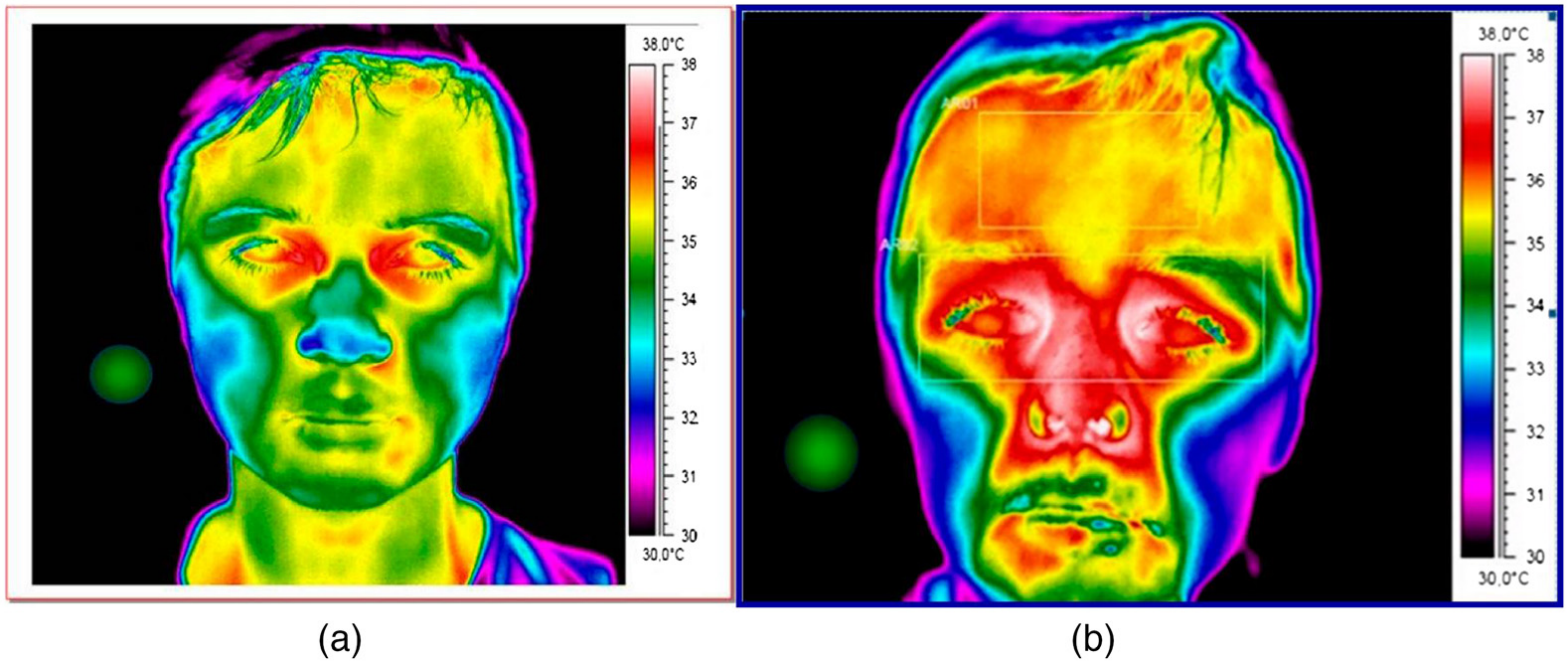
Thermal image: (a) without fever and (b) with fever.^29^

IRT, a non-contact and real-time thermometer, has become widely used in various clinical applications including oncology, dermatology, vascular disorders, and for fever screening.^24^^–^^26^ For example, inflammation from skin defects such as tungiasis was screened and quantified using IRT to measure inflammation-induced changes in skin temperature.^24^ In addition, thermal patterns of diabetic patients with and without vascular complications were compared using IRT.^27^ Unlike traditional thermographic instruments, IRT provides a live thermal map over a wide anatomical region, which enables analysis of body temperature distribution including any hot or cold spots.^28^ As a demonstration, Fig. 2 shows a thermal face map of an individual with and without fever.

IRT uses the properties of human infrared emission to capture thermographic information. All objects with a non-zero Kelvin temperature emit infrared radiation at wavelengths between 0.75 and 1000  μm. Human skin emissions range from 2 to 20  μm in wavelength, peaking around 10  μm. The intensity of this infrared radiation can be mathematically translated to surface body temperature, a clinically important parameter.^24^^,^^25^^,^^30^ Together with the advantages of being a non-contact and real-time sensing technique with a wide anatomical region measurement, IRT emits no harmful radiation, which makes it suitable for public and long-term use.^31^ However, it requires a controlled environment, in which ambient temperature and humidity are maintained within specific ranges. Wide variance of such conditions can result in inaccurate thermographic readings.^26^ Additionally, IRT-based thermal screening is affected by other factors including medication, cosmetics, pregnancy, and physical activity.^25^^,^^32^

#### 1.3.1 IRT: instrumentation

First generation thermal camera systems, developed in the 1970s, used a single infrared detector and two scanning mirrors to digitally generate a thermal image. Second generation thermal imagers, developed in the 1980s, introduced larger arrays of detectors (up to 64×64).^32^ These systems used time-delay integration for higher image quality.^33^ Current systems are comprised of an infrared sensor, image analysis hardware, and a real-time display monitor.^25^ Images captured by the infrared camera are converted into electrical signals and processed using a workstation or on-chip image processing hardware for real-time display and analysis.^30^ Upgrades to infrared sensor and camera technology have allowed for much larger two-dimensional detection arrays, upward of 1024×1024 elements, and improved optomechanical technology has permitted for the scanning of larger anatomical regions.^30^^,^^33^ The advance of microbolometric detectors, which require no cooling, has allowed for smaller and more lightweight IRT systems that can start up quickly. These advancements have also resulted in a significant improvement in noise equivalent temperature difference (NETD) of IRT systems over the last 20 years. Older IRT systems reported NETD in the 70- to 150-mK range, whereas current systems, including compact low-cost technologies, report NETD of <50  mK.^30^^,^^34^^,^^35^

#### 1.3.2 IRT: data analysis and image processing

IRT data processing involves digitizing the measured signal from the infrared camera, processing the data, and extracting body temperature. Digitization includes the transformation and quantification of infrared radiation into a spatial infrared image. These steps are dependent on the type of detector used, detector array size, and sampling rate of the system.^31^ Several pre- and postprocessing algorithms have been implemented in IRT systems to improve image quality. IR image preprocessing improves uniformity within images by correcting for fluctuating light conditions.^36^ Filters have been applied to thermal images to minimize noise and reduce blurring. In addition, operations including background subtraction and time derivative calculation of thermal data have been used to increase the accuracy of data.^25^ Current research to improve IRT data processing includes asymmetry analysis of thermal images, smart image enhancement and restoration algorithms, and automatic feature detection and classification algorithms.^36^ Effectively and efficiently detecting and segmenting anomalies in thermal images can be difficult to do with the sheer number of co-founding factors that can reduce the accuracy of the IRT images. Hierarchical clustering-based segmentation (HCS) is one such method of identifying features within IRT images that can be quite noisy.^37^ The advantage of this process compared to other segmentation algorithms is that HCS employs a hierarchy of thresholding rather than a single threshold value when identifying boundaries of regions of interests within IRT images.^37^ This minimizes the loss of useful data during the processing stage of IRT images. HCS is a versatile segmentation process that can be applied to IRT images from a wide variety of sources with minimal tuning.^37^^–^^39^ Examples include organic materials like plants in the environment and variations in body temperature postsurgery or inorganic cases such as differentiating different types of window materials using IRT imaging. To extract a temperature from processed infrared data, the physiological target radiation must be isolated from total radiation received by the camera, which also includes radiation from the atmosphere and surroundings.^31^

#### 1.3.3 Effects of environmental conditions on IRT results

Several studies have been conducted to investigate the effects of various experimental parameters on thermal measurement accuracy. The United States Food and Drug Administration^40^ quantitatively compared two moderately priced commercial IRTs in various environmental conditions. The study found that the temperature, humidity, and type of data processing methods significantly affected IRT results.^26^ Ring et al.^23^ noted that the laboratory environment must be thermally stable. Research has also shown that factors such as system stability and drift, curvature of the anatomical region, precision of the IRT system, secondary infrared light sources, and participant use of cosmetics and antiperspirants can affect the accuracy of collected data.^25^^,^^26^

#### 1.3.4 State-of-the-art commercial and research systems

Many commercial and research IRT systems have been developed. Commercial manufacturers include FLIR Systems Inc., which has produced numerous infrared thermal cameras with varying resolution, portability, and analysis capabilities. Seek thermal has created miniaturized, low-power IRT systems including ones that can be attached to a smartphone or tablet camera, transforming it into a thermal imager. Table 2 lists specifications of two typical IR cameras, which have been used in many studies.^41^^,^^42^ Research systems adapted traditional IRT techniques and combined them with other clinical sensing modalities with software-based analysis tools. Several high-resolution IRT systems have been developed, including a three-dimensional IRT system in 2017 that can simultaneously obtain true-color images of the physiological region.^24^^,^^43^ A computer-assisted video thermography system has been developed that reduces subjectivity in interpretation of thermographic images by analyzing them using a software-based algorithm.^44^ In addition, a multi-modal system has been developed using IRT, a blood-flow meter, and microwave radar to provide more robust screening of suspected respiratory infection patients.^45^

**Table 2:** Specifications of two typical IR cameras.

Manufacturer	FLIR Systems, Inc.^41^	Optotherm^42^
Product	T500 series	Thermoscreen
Detector type	Uncooled microbolometer	Uncooled amorphous silicon
Pixel pitch (μm)	17	17
Array size	464×348	640×480
Thermal sensitivity/NETD	<30 mK at 30°C	<40 mK at 30°C
Accuracy	±2°C	±0.3°C between 30 and 40°C±1°C otherwise
Operating temperature range	−15°C to 50°C	15°C to 35°C
Frame rate (Hz)	30	60
Working F-number	1.1 with 42-deg lens	1.02

## 2 Materials and Methods

A literature review was performed in Google Scholar, PubMed, and ScienceDirect to search for studies evaluating IRT screening from 2002 to present. The following key words were utilized: mass screening; fever; fever screening, thermometer, digital thermometer, IRT, thermal camera, thermogram, IRT sensitivity and specificity, IRT instrumentation, IRT data analysis, IRT generations, IRT components, infectious disease, respiratory infection, respiratory symptoms, infectious disease diagnosis, infectious disease detection, flu, COVID-19, SARS, MERS, Ebola, Influenza, Dengue, Zika, The Black Death, Smallpox, and HIV. The identified studies for the evaluation of mass screening using IRT were then selected based on the availability of information addressing total number of screened individuals, detected individuals, patients, device sensitivity, and specificity. Reported studies not containing the listed information were excluded from the review. Additional literature searches sought to identify literature specifically evaluating IRT in comparison to traditional measurements of core body temperature and comparing different IRT device components and instrumentation to each other through device sensitivity and specificity measurements. Finally, a search was done to locate studies assessing the benefit of implementing measurements of extra vital signs for the detection of infectious diseases from 2002 to present. The key words utilized were similar to the above list with inclusion of heart rate and breathing rate. Of the identified papers, studies reporting the number of subjects (patients and controls), device sensitivity, device specificity, PPV, and NPV were included. The identified literature not reporting these values were excluded.

For literature evaluating the effectiveness of IRT, six studies were identified comparing the measurements of surface and core body temperature for the detection of illness: one aimed at assessing wireless dermal thermometers as a replacement for invasive measurements,^46^ four studies aimed at studying the variations in skin temperature to variables unrelated to illness,^20^^,^^47^^–^^49^ and one analyzing the correlation between tympanic membrane temperature and the temperature at various facial regions.^50^^,^^51^ Additionally, four studies were found that investigated IRT accuracy using the forehead for the thermographic region of interest: one analyzing IRT for mass blind screening in Singapore,^52^ one assessing IRT during the H1N1 pandemic in Hong Kong,^53^ one from Taiwan assessing digital infrared thermal imaging (DITI) to conduct screenings on SARS patients,^30^ and one study in France evaluating IRT accuracy for fever screening.^54^ One study in the United States was found comparing the capabilities of three different infrared thermal detection systems and was included in the review.^34^ For literature assessing the locations for mass fever screenings, eight articles were identified; two aimed at the analysis of airport fever screening for Dengue in Taiwan;^12^^,^^13^ two aimed at fever screening at sea ports in Australia and Singapore;^14^^,^^15^ and two aimed at fever screening at border crossings in Singapore.^15^^,^^16^ Two studies were focusing on the effectiveness of IRT in screening COVID-19 patients in Pakistan and United States.^55^^,^^56^ Additionally, three studies were identified assessing the effectiveness of screening to delay local transmission.^17^^–^^19^ Several studies were also included that analyzed the impact of a controlled environment on screening accuracy.^21^^,^^57^^–^^59^ For literature evaluating the efficiency of IRT screening for the detection of febrile international travelers, seventeen studies were identified: one aimed at detecting SARS in Canada;^60^ four studies for Dengue detection in Taiwan;^12^^,^^13^^,^^61^^,^^62^ five for Influenza in New Zealand,^63^^,^^64^ Japan,^65^^,^^66^ and Australia;^67^ and three for Ebola virus disease (EVD) in the USA, Australia, and the UK,^68^ and Sierra Leone;^69^^,^^70^ one for MERS in Indonesia;^71^ and three for COVID-19 in multiple countries.^72^^–^^74^ For literature evaluating the detection of infectious diseases with measurements of vital signs, three studies were identified containing the required information for inclusion: two utilizing CMOS camera that was equipped with IRT;^75^^,^^76^ two utilizing Doppler blood-flow meter, 10-GHz microwave radar, and thermography;^77^^,^^78^ and one utilizing radar, finger-tip photoreflector, and thermography.^79^

## 3 Results

### 3.1 Specificity and Sensitivity of IRT in Detecting Fever

Several studies have been conducted to investigate the efficacy of IRT as a tool for fever detection, which used the forehead as the thermographic region of interest. The results of these studies are summarized in Table 3. A 2004 study in Singapore by Ng et al.^52^ analyzed the capability of IRT for mass blind fever screening of 310 individuals and found a sensitivity and specificity of 89.6% and 94.3%, respectively. They concluded that IRT can serve as the first line tool for fever screening if calibrated for outdoor environmental factors. A 2005 study in Hong Kong by Ng et al. compared non-contact infrared forehead temperature (NIFT) measurement to tympanic temperatures in 500 children. The study found that NIFT had a sensitivity and specificity of 89.4% and 75.4%, respectively, of detecting fever using the cutoff point determined by tympanic temperature measurement.^53^ In Taiwan, Chiu et al.^30^ used a DITI system to conduct mass screening of suspected SARS patients. A total of 993 suspected febrile patients were screened and the study found a sensitivity and specificity of 75% and 99.6%, respectively. A 2008 study in France evaluated the diagnostic accuracy of IRT for fever screening and tested 2026 patients in different groups based on predicted tympanic temperature. Sensitivity and specificity of their device were found to be 82% and 77%, respectively.^54^ In the United States, Nguyen et al.^34^ compared three different infrared thermal detection systems, the FLIR ThermoVision, A20M, the Opto Therm Thermoscreen, and the Wahl Fever Alert Imager HSI20000S, to assess their screening capabilities. More than 2000 patients were tested with each system. The sensitivity and specificity of each device were as follows: FLIR (90.0%, 80.0%), OptoTherm (91.0%, 86.0%), and Wahl (80.0%, 65.0%).^34^ In total, these values range from 75.0% to 91.0% for sensitivity and 65.0% to 99.6% for specificity. Variation in these values results from study-to-study differences in IRT device, experimental conditions, and threshold used to classify a successful measurement.

**Table 3:** Summary of studies using IRT to detect fever.

Author, year, country	Sample size	Sensitivity (%)	Specificity (%)
Ng et al., 2004, Singapore^52^	310	89.6	94.3
Ng et al., 2005, Hong Kong^53^	500	89.4	75.4
Chiu et al., 2005, Taiwan^30^	993	75.0	99.6
Hausfater et al., 2008, France^54^	2026	82	77
Nguyen et al., 2010, United States (OptoTherm Thermoscreen, FLIR ThermoVision A20M, Wahl Fever Alert Imager HSI20000S)^34^	2507	91.0	86.0
	2515	90.0	80.0
	2061	80.0	65.0
Khan et al., 2020, Pakistan^55^	538	13.61	97.95
Zhou et al. 2020, United States (A325sc, FLIR Systems Inc., 8640 P-series, Infrared Cameras Inc.)^56^	544	85	94
	540	94	89

### 3.2 Diseases and Public Screening by Infrared Thermography

Entry/exit screening was performed during different pandemics including the influenza pandemic (H1N1) in 2009, MERS in Saudi Arabia in 2012, EVD in West Africa in 2014, SARS in Australia, Canada, and Singapore in 2003, and the most recent COVID-19 outbreak in China in 2019.^63^^,^^64^ However, very low sensitivity and specificity were reported.^25^ Health questionnaires, interviews, and careful examination of the traveler were suggested as alternatives because these provide a more extensive picture of the risk of someone having an infectious disease.^80^ Also social awareness, school closure, home quarantine, and social distancing are reported as more important variables in the disease transmission than entry/exit screening.^81^

Cowling et al., after the influenza type A (H1N1 or swine flu) outbreak, stated that entry screening of travelers may lead to short-term delay (1 to 2 weeks) in local transmission of influenza virus. In that work, they considered and reported on the results of 35 nations with more than 100 H1N1 positive cases reported to the World Health Organization.^81^ In 2017, Sun et al.^82^ performed an IRT evaluation for detecting febrile international travelers entering Japan at Nagoya Airport (2003 to 2004) and Naha International Airport (2005 to 2009) after the SARS pandemic. They reported several limitations with the accuracy of IRT, such as taking antifebrile medications that affect the efficiency of IRT with a rapid modification of the body temperature.

### 3.3 Mass, Blind Screening: Sensitivity and Specificity

After the SARS pandemic in 2003, many countries established a mass screening system, usually a non-contact thermography system to detect fever in international airports. These systems have been employed to screen passengers at the entry and/or exit gate to prevent entry of the virus into a country and/or spreading the virus to other countries. Seventeen studies were identified that reported the efficiency of the screening system in detecting SARS (one study), dengue (four studies), influenza (five studies), EVD (three studies), MERS (one study), and COVID-19 (three studies). The summary of the total screened passenger, detected fever, detected patients, not detected patients, sensitivity, specificity, PPV, and NPV of these studies is presented in Table 4. The detection sensitivity was as low as 0% in SARS, Ebola, influenza, MERS, and COVID-19 detections but was higher in Dengue, Chikungunya, and Zika detection.

**Table 4:** Mass screening of infectious disease in the airport.

Country	Disease, year	Total	Detected	Patient	Patient not detected	Sensitivity (%)	Specificity (%)	PPV (%)	NPV (%)
Canada^60^	SARS, 2003	1,172,986	2889	0	—	0	—	—	—
Taiwan^13^	Dengue, 2003 to 2004	8,000,000	22,000	40	25	65.8	—	—	—
Taiwan^61^	Dengue, 2003 to 2007	—	—	244	298	45	—	30.5 to 62.6	—
Taiwan^12^	Dengue, 2007	12,508,621	11,118	72	107	40.2	99.96	1.28	100
	2008	12,202,392	12,158	100	125	44.4	99.96	2.03	100
	2009	12,499,365	12,286	108	95	53.2	99.97	2.9	100
	2010	14,837,391	12,553	126	175	41.86	99.97	3.22	100
New Zealand^63^	Influenza, 2008	5274	1275	30	—	83 to 87	11 to 39	2.0 to 2.8	—
Australia^67^	H1N1 Influenza, 2009	625,147	5845	3	45	6.67	99.1	0.05	—
New Zealand^64^	H1N1 Influenza, 2009	456,518	406	4	69	5.80	—	—	—
Japan^65^	H1N1 Influenza, 2009	471,733	805	10	141	6.60	—	—	—
Japan^66^	H1N1 Influenza, 2009 to 2010	9,140,435	930	0	—	0	—	—	—
Taiwan^62^	Dengue, 2013	19,072,276	12,924	115	148	44	—	—	—
	Chikungunya			17	29	59	—	—	—
	Dengue	21,707,379	15,280	118	127	48	—	—	—
	Chikungunya			4	7	57	—	—	—
	Dengue	23,601,215	17,779	155	210	42	—	—	—
	Chikungunya			4	0	100	—	—	—
	Zika	21,083,404	21,721	5	8	38	—	—	—
	Dengue			130	185	41	—	—	—
	Chikungunya			4	4	50	—	—	—
US^68^	Ebola, 2014	1993	86	0	—	0	—	—	—
Australia^68^		122	6	0	—	0	—	—	—
UK^68^		3388	130	0	—	0	—	—	—
Guinea, Liberia, and Sierra Leone^69^	Ebola, 2014 to 2016	300,000	—	0	4	0	—	—	—
Sierra Leone^70^	EVD, 2014 to 2016	166,242	10	0	2	0	—	—	—
Indonesia^71^	MERS, 2015	28,197	15	0	—	0	—	—	—
India^72^	COVID-19, 2020	1,587,034	151	0	—	0	—	—	—
Multiple countries^73^	COVID-19, 2020	—	—	14	257	5.2			
US^74^	COVID-19, 2020	766,044	278	9	14	39			

### 3.4 Combination of IRT with Other Techniques for Screening Infectious Disease

Along with temperature, several groups have suggested the measurement of extra vital signs including heart rate and respiratory rate.^75^^–^^78^ They have claimed that since the inflammation not only causes an elevation in body temperature but also increases to heart and respiration rates, inclusion of these multiple vital signs will improve screening accuracy. In 2010, Matsui et al.^75^ employed laser doppler-flow meter to obtain heart rate, 10-GHz microwave radar to detect breathing rate, and thermography to measure skin temperature on 92 subjects (57 patients with H1N1 influenza and 35 controls). By applying linear discriminant analysis on the multimodal data, they achieved 88% sensitivity and 89% specificity. After that, Yao et al.^78^ used other classification algorithms, including support vector machine, k-nearest neighbors, and logistic regression, on the same data sets to improve the sensitivity to 93%. Similarly, high sensitivity (97.1% and 87.5%) and specificity (81.3% and 100%) were obtained in other studies when multimodal signals were measured.^77^^–^^79^ Notably, Sun et al. claimed that the inclusion of heart rate and respiration rate enhances the sensitivity by 18.8% compared to when temperature alone was used. Table 5 summarizes the techniques used, number of subjects, sensitivity, specificity, PPV, and NPV of five studies measuring multiple vital signs to classify patients with influenza from the healthy control.

**Table 5:** Summary of five studies using different techniques to measure heart rate, respiration rate, and temperature in patient with influenza and healthy controls.

Paper	System	Number of subjects		Sensitivity (%)		Specificity (%)	PPV (%)	NPV (%)
		Patient	Control	Fever-based	All			
Sun, 2017^75^	CMOS camera equipped with IRT	16	22	68.70	87.50	100	100	91.70
Negishi, 2019^76^		12	13	—	—	—	—	—
Matsui, 2010^77^	Doppler blood-flow meter, 10-GHz microwave radar, thermography	57	35	—	88	89	93	82
Yao, 2016^78^		57	35	—	93	—	—	—
Sun, 2015^79^	Radar, finger-tip photoreflector, thermography	35	48	—	97.10	81.30	79.10	97.50

## 4 Discussion

Though IRT is capable of real-time, non-contact measurement of body surface temperature over a wide anatomical area, its measurement accuracy depends heavily on environmental parameters. Additionally, body surface temperature is not always a reliable surrogate for the core body temperature that is affected by infection. Rectal and esophageal temperature are reported to be the most reliable and easily accessible body sites to obtain core body temperature,^21^ but they are invasive and not appropriate sites for mass screening. On the other hand, sublingual, axillary, inguinal sites, auditory canal, and forehead are more common sites for measuring temperature using clinical thermometers, but they do not reflect the true core body temperature. In this regard, modern IRT suggests capturing thermograms of the human face non-invasively and using various algorithms to compensate for the underestimated core body temperature. Here the question would be which site(s) on the human face is the best representative of the change in core temperature. Ring et al.^83^ suggested using canthus measurement as a more reliable measurement of the core temperature. Although it is not difficult to detect an increase in body temperature through canthi, there is a complicated relationship between this temperature and the real core temperature. Other studies focused on forehead or auditory meatus temperature for easier measurement, but limitations are reported.^84^ Ultimately, the lack of scientific data showing the relationship between human head (face) temperature and core body temperature remains a challenge to be addressed.

Another challenge with mass screening using IRT is the inability to detect the fever development in incubating or asymptomatic patients during early or late stages. An infected individual might not present with a fever during the incubation period. Thus the febrile screening system is not able to capture the case. Additionally, a normal body temperature will be for previously infected individual who are already on fever suppressant. On the other hand, there might be other conditions for a reported high fever that are not due to a viral infection. Some medications such as hormone treatments, severe trauma and injury, and other medical conditions or pregnancy can cause an elevated body temperature. In these cases, a reported high fever with IRT might be mistaken as an infection.

The presence of a fever due to an infection depends on various parameters such as age, the immune system status, the inflicting virus, and the disease stage. A study in Finland examined patients with laboratory-confirmed influenza and found fever present in 89% of children younger than 13 years old.^85^ In another work, Chughtai et al.^86^ stated that fever is less common in adults with confirmed respiratory infections than in children, reporting that 75% of adults showed no fever. Carrat et al. compared fever in adults with different types of influenza and found that the prevalence of fever in those inflicted with influenza differs between viral strains (H3N2: 95.2%, H1N1: 77.5%).^87^ Moreover, it is reported that some infectious respiratory diseases have only respiratory symptoms.^88^

Camera quality plays an important role in thermography. Low camera resolution, poor focus, and placing the camera too far from the subject may cause inaccurate measurement. Training personnel, regularly testing cameras, and following essential protocols may help with reliability and reproducibility of the outcomes of the technique.^21^

Although fever is a primary symptom in the majority of infectious diseases, many studies have demonstrated that measuring body temperature alone is insufficient in detecting infections.^12^^,^^13^^,^^60^^–^^70^^,^^75^^–^^79^ As has been suggested previously, in addition to body temperature, heart rate and respiratory rate are the two crucial vital signs needed to be monitored.^75^^–^^79^ Additionally, since many infectious diseases, especially coronavirus related infections, cause SARS, monitoring breathing related parameters may enhance sensitivity and specificity of disease screening.

## 5 Conclusion

The COVID-19 outbreak and resultant efforts in preventing disease transmission has raised the alarm to re-examine screening methods for infectious diseases. High temperature, a typical indicator of an infection, is the only parameter considered for mass screenings at airports and borders during an epidemic. Since the 2003 SARS outbreak, infrared thermal cameras have been mounted at airports in countries such as Canada, Taiwan, and Australia.^13^^,^^60^^,^^68^ Several groups studied the efficacy of mass fever screening using thermal cameras in those airports, but they have not found reliable outcomes in detecting febrile individuals using these systems. Low camera quality, very low sensitivity to true body temperature, and inability to detect asymptomatic patients were reported as the main reasons that thermal cameras alone are not reliable.^21^ It has been suggested that other vital physiological parameters should be monitored as extra indicators of an infection to obtain more consistent results from mass screening.^75^^–^^79^ In addition to an elevated body temperature, a patient with respiratory infectious disease such as COVID-19 experiences a change in tissue oxygenation, cardiovascular, and respiratory functions. Therefore, there is an urgent need to develop a new technique capable of rapidly screening all these signals and integrating the measured parameters into new metrics for early detection of viral infections. With the advent of wireless technologies, this approach, ideally, can lead to the development of sensors with point-of-care home-accessible capabilities to manage the growing number of infected patients staying in home quarantine, eventually alleviating the burden on the healthcare system.
